# Effects of calcium supplementation on the prevention of preeclampsia: an umbrella review of systematic reviews and meta-analyses

**DOI:** 10.3389/fmed.2025.1434416

**Published:** 2025-03-05

**Authors:** Henok Kumsa, Esuyawkal Mislu, Mulugeta Wodaje Arage, Biruk Beletew Abate, Moges Beriye, Mihretab Mehari Reda, Nigus Bililign Yimer

**Affiliations:** ^1^School of Midwifery, College of Health Sciences, Woldia University, Woldia, Ethiopia; ^2^School of Nursing, College of Health Sciences, Woldia University, Woldia, Ethiopia; ^3^School of Population Health, Curtin University, Bentley, WA, Australia; ^4^School of Medicine, College of Health Sciences, Woldia University, Woldia, Ethiopia; ^5^Department of Midwifery, College of Health Sciences, Mekelle University, Mekelle, Ethiopia

**Keywords:** preeclampsia, calcium supplementation, umbrella review, systematic review, meta-analysis

## Abstract

**Background:**

Preeclampsia is the leading cause of maternal and fetal morbidity and mortality. Calcium supplementation has been considered a potential intervention to reduce the risk of preeclampsia. This umbrella review aims to summarize the effects of calcium supplementation in the prevention of preeclampsia based on existing systematic reviews and meta-analyses studies.

**Methods:**

A systematic search of electronic databases, such as MEDLINE, Web of Science, SCOPUS, and the Cochrane Library, was conducted from inception to 30 December 2023. The methodological quality of the included studies was assessed using the revised version of the Assessing the Methodological Quality of Systematic Reviews (AMSTAR 2) tool. A random-effects model was used to estimate the effect of calcium supplementation on preeclampsia. Heterogeneity among included studies and publication bias were assessed using the I^2^ statistic and the Egger’s test, respectively.

**Results:**

Calcium supplementation reduced the risk of preeclampsia by 47% (RR: 0.53, 95% CI: 0.42, 0.68) with a considerable level of heterogeneity (I^2^ = 84.39%). Our subgroup analyses revealed that the risk of preeclampsia was significantly lower in high-risk pregnancies that received calcium supplementation (RR: 0.35, 95% CI: 0.26, 0.47), indicating a 65% risk reduction. In comparison, low-risk pregnant women who received calcium supplementation experienced a 33% risk reduction (RR: 0.67, 95% CI: 0.59, 0.77). Furthermore, the effects of calcium supplementation were more pronounced in women from developing countries compared to those from developed countries.

**Conclusion:**

This umbrella review provides a summary of the evidence supporting the use of calcium supplementation to reduce preeclampsia. Incorporating calcium supplementation into antenatal care interventions may help to reduce the burden of preeclampsia and improve maternal and fetal outcomes. Further studies are needed to explore the impact of baseline calcium levels, optimal dosage, timing, and routes of supplementation to effectively decrease the incidence of preeclampsia.

## Background

Preeclampsia is a pregnancy-specific multisystem complication characterized by high blood pressure, proteinuria, and organ damage that poses a significant risk to both the mother and the fetus. Its incidence varies across regions, affecting 2–5% of pregnancies worldwide (1). Evidence suggests that the burden of preeclampsia is greater in developing countries, with a reported rate of 2.8% of live births, which is seven times higher than the rate in developed countries (0.4%) (2). Preeclampsia is one of the leading causes of maternal and perinatal morbidity and mortality worldwide, accounting for up to 29,000 maternal deaths annually (3). Calcium supplementation has emerged as a topic of interest and investigation for the prevention of preeclampsia (4, 5).

Calcium plays a crucial role in muscle contraction, maintaining water balance, and regulating blood pressure. Insufficient calcium levels trigger the release of parathyroid hormone, resulting in elevated intracellular calcium concentrations in vascular smooth muscle cells. This leads to vasoconstriction and high blood pressure (6). Consequently, maintaining adequate calcium levels ensures vascular stability, thereby reducing the risk of hypertension (6). Moreover, dietary calcium supplementation has been observed to decrease renin levels in the blood, although it does not completely eliminate them. In addition, the renin-angiotensin-aldosterone system, which plays a pivotal role in regulating blood pressure, is influenced by the acute activation of calcium-sensing receptors in juxtaglomerular cells due to high extracellular calcium concentrations. Taken together, these factors contribute to the preventive effect of calcium against hypertension (7, 8).

During pregnancy, adequate blood calcium levels are associated with positive maternal–fetal health outcomes and a reduced risk of maternal and fetal cardiovascular disease (9). However, the demand increases due to a combination of physiological, metabolic, and hormonal factors in a period of pregnancy (10, 11). Calcitonin hormone, which is responsible for calcium deposition in the bones, is also elevated during pregnancy (12). Furthermore, vitamin D, which plays a crucial role in calcium metabolism and bone health, can also be affected during pregnancy (13). These conditions indicate the need for a relatively high calcium intake during pregnancy. However, insufficient dietary calcium intake can impair the body’s ability to maintain calcium balance, potentially causing maternal calcium deficiency and complications, such as disrupted vascular tone regulation, preterm birth, and fetal growth restriction (13, 14). This is particularly concerning in mothers who have a low dietary calcium intake (5). In 2015, the World Health Organization (WHO) recommended calcium supplementation for pregnant women (15) and has subsequently updated its guidelines to emphasize the potential benefits of calcium in the prevention of preeclampsia (15–17). However, a review of low- and middle-income countries revealed that the average calcium intake was less than 900 mg/day (18). Subsequent reviews in 2004 and 2018 indicated that dietary calcium intake remained at approximately 600 mg/day in these regions, with no significant improvement, compared to over 1,000 mg/day in high-income countries, resulting in a difference of approximately 300 mg/day mean calcium intake (19, 20).

There is limited and inconsistent evidence regarding the effectiveness of calcium supplementation in preventing preeclampsia. A systematic review and meta-analysis of four randomized trials that included 7,272 women reported that calcium supplementation led to an 11% reduction in the incidence of preeclampsia (10). Another review conducted by Christina Oh et al. (21) indicated a 70% reduction in the risk of preeclampsia (21).

Therefore, this umbrella review aims to summarize and address the inconsistencies found in systematic reviews and meta-analyses on the effectiveness of calcium supplementation in preventing preeclampsia. Furthermore, this review aims to consolidate current knowledge, identify potential sources of heterogeneity, and provide valuable insights into the effectiveness of calcium supplementation in reducing the burden of preeclampsia, ultimately guiding clinical practice and informing future research endeavors.

## Methods

### Design

This umbrella review was conducted to evaluate the effects of calcium supplementation on the prevention of preeclampsia using existing systematic reviews and meta-analyses studies. The review process adhered to the Preferred Reporting Items for Systematic Reviews and Meta-analyses (PRISMA) guidelines (22). The target population (P) comprised pregnant women at risk of developing preeclampsia, the intervention (I) was calcium supplementation, the comparator (C) was pregnant women who did not receive calcium supplementation, and the outcome (O) was preeclampsia, defined as a pregnancy-specific illness characterized by the onset of hypertension after 20 weeks of gestation, with or without proteinuria (23). This review was registered with PROSPERO (CRD42024547701).

### Search strategy

Using a combination of Medical Subject Headings (MeSH) and keywords, we systematically searched MEDLINE via PubMed, Embase, Web of Science, Scopus, the Cochrane Database of Systematic Reviews, the PROSPERO register, and the Google Scholar web search engine. MeSH terms and keywords included ‘preeclampsia,’ ‘hypertensive disorders during pregnancy,’ ‘pregnancy-induced hypertension,’ ‘calcium,’ ‘Ca++,’ ‘supplementation,’ ‘systematic review,’ and ‘meta-analysis.’ The search period was from inception to 30 December 2023. Additionally, citations of the eligible studies were manually searched. The detailed search strategy is included in the Supplementary Table S1.

### Study inclusion criteria

#### Types of studies

This umbrella review included all systematic reviews and meta-analyses studies that assessed the effects of calcium supplementation in the prevention of preeclampsia.

#### Types of participants

The participants included in this review were women who received calcium supplementation during their pregnancy, regardless of whether the pregnancy was a singleton or multiple gestation. A pregnant woman considered to have an unknown status was defined as one who received calcium supplementation despite her risk for preeclampsia, her country of residence and her baseline status. In contrast, pregnant women participating in the study were categorized into two subgroups based on their risk profile. The high-risk group consisted of pregnant women who exhibited calcium deficiency, a history of gestational hypertension or preeclampsia, a positive roll-over test, a positive angiotensin-sensitivity test, or other high-risk factors as defined in the original study. The low-risk group included healthy pregnant women with a lower risk of developing these conditions (5). Additionally, baseline calcium intake levels were categorized as low if they were below 900 mg/day and adequate if they were 900 mg/day or above (5). Moreover, for the analysis, a high dose was defined as ≥1 g of elemental calcium per day, a medium dose was defined as 0.6–1.5 g, and a low dose was defined as <1 g of elemental calcium per day.

#### Types of interventions

Studies were eligible if they compared calcium supplementation during pregnancy with a placebo group, no treatment. The included studies considered different risk factors for preeclampsia, dietary intake, dosage variations, and the economic status of the countries involved.

#### Outcomes of interest

The primary outcome of interest was the incidence of preeclampsia.

#### Setting

This umbrella review included studies conducted globally without any geographical limitations.

#### Publication condition

This review included only published articles in English that had full texts and reported the effect of the outcome of interest.

### Selection and data extraction

The retrieved systematic review and meta-analysis studies were exported into EndNote software to eliminate any duplicate studies. Two independent reviewers (HK and NBY) retrieved records from the databases and then screened the articles based on their titles and abstracts. The records retained during this screening phase underwent full-text assessment by the two reviewers. Disagreements between the two reviewers were resolved through consensus and the involvement of a third reviewer. A data extraction form was used to extract information from the included studies. The form includes author, year of publication, sample size (number of primary studies), intervention and control group sample sizes, effect estimates with confidence intervals, dose levels, the economic status of the countries, calcium dietary status, and risk status of women. Furthermore, the data extraction process in Microsoft Excel involved transforming each factor’s relative risk (RR) to its logarithmic form.

Additionally, the upper and lower confidence intervals were also log-transformed. The standard error (SE) of these confidence intervals was then calculated using the formula SE = (logUCL—logLCL) / 3.92, where logUCL and logLCL represent the logarithmic upper and lower confidence limits. The log-transformed RR and the SE of their corresponding confidence intervals were utilized in the pooled RR estimation to estimate effect sizes.

### Quality assessment

The methodological quality of the included studies was assessed using the revised version of the Assessing the Methodological Quality of Systematic Reviews (AMSTAR 2) tool (24). AMSTAR 2 has 16 method-related questions that serve as a critical appraisal tool for systematic reviews and meta-analyses that include randomized or non-randomized studies. Two independent reviewers, EM and MW, critically appraised the included studies using the checklists. As suggested in AMSTAR 2, we rated the confidence in the results of the included reviews as follows: high (no or one non-critical weakness), moderate (more than one critical weakness but no critical flaws), low (one critical flaw with or without non-critical weaknesses), and critically low (more than one critical flaw with or without non-critical weaknesses). The Supplementary Material includes the quality assessment of the studies included in this umbrella review (Supplementary Table S2). The quality of the primary studies included in each of the research syntheses was rigorously evaluated to ensure their robustness and reliability. The list of primary studies in the meta-analyses is in the Supplementary Table S3.

### Data synthesis and statistical analysis

After extracting the data using Microsoft Excel, we imported them into STATA version 17.0 for further analysis. This allowed us to perform statistical tests and sensitivity analyses and to apply meta-analytic techniques to ensure robust and reliable results. The findings of the included studies were presented quantitatively and qualitatively to provide comprehensive evidence about the current use of calcium supplementation and its potential effects on the reduction of preeclampsia. A random-effects model meta-analysis was conducted to summarize the effects of calcium supplementation on the reduction of preeclampsia. The estimates were reported as relative risks with corresponding 95% confidence intervals.

Heterogeneity between studies was assessed using the I^2^ statistic. The I^2^ statistic describes the percentage of variation across different studies due to heterogeneity (25). We used the Q test to estimate I^2,^ and heterogeneity was classified by I^2^. The findings of the I^2^ test were classified as low (25% and below), moderate (26–50%), high (51–75%), and very high (above 75%) (25). If there was evidence of heterogeneity, we used a subgroup analysis using different characteristics of the included studies, such as preeclampsia risk status, baseline dietary status, and dosage of calcium supplement. Publication bias was assessed using a funnel plot and an Egger’s regression test. An asymmetric funnel plot and an Egger’s test with results lower than 0.05 were considered indicative of publication bias. Sensitivity analyses were performed to evaluate the influence of individual studies on the overall pooled effect estimate. This involved systematically removing one study at a time from the meta-analysis and recalculating the overall effect size to observe any significant changes.

## Results

**Figure 1:**
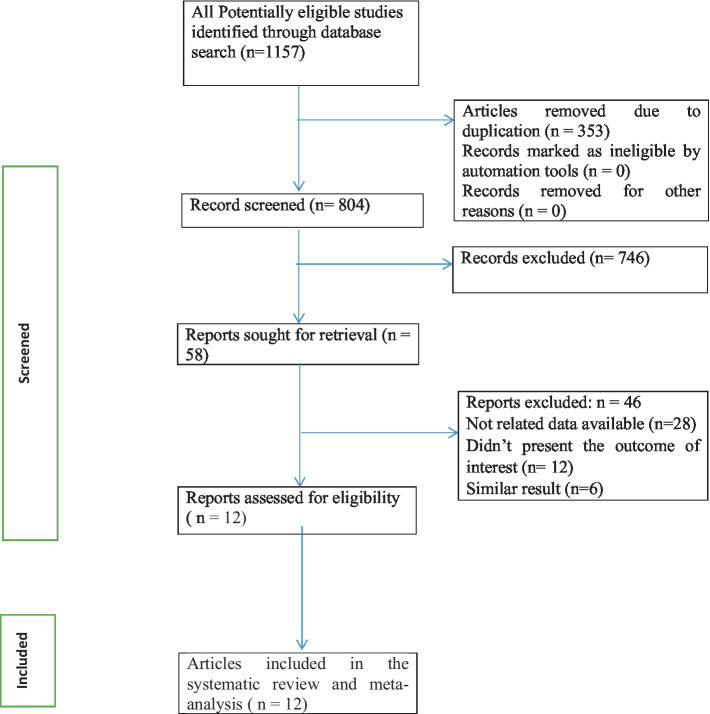
Flow chart of study selection for an umbrella review of systematic reviews and meta-analyses of the effect of calcium supplementation on prevention of preeclampsia in calcium supplemented group compared to placebo.

This umbrella review of systematic reviews and meta-analyses includes published studies on calcium supplementation for the prevention of preeclampsia. The included studies were systematic reviews and meta-analyses published from 1996 to 2022. A total of 1,157 records were retrieved through electronic searches, and 12 studies were included to estimate the effects of calcium supplementation in the prevention of preeclampsia (Figure 1).

### Characteristics of included studies

In the systematic reviews and meta-analyses, the number of primary studies included ranged from a minimum of 3 to a maximum of 29. The minimum and maximum sample sizes in these included studies were 1,324 participants (660 in the experimental group and 664 in the control group) and 27,765 participants (13,896 in the experimental group and 13,869 in the control group), respectively. With the exception of the review by Christina Oh et al. (21) which included quasi-experimental studies, the remaining reviews included only randomized controlled trials in their meta-analyses. Eight systematic reviews and meta-analyses reported the effects of calcium supplementation on the reduction of preeclampsia, regardless of dose, duration, baseline dietary intake, and risk status for preeclampsia. Additionally, systematic reviews and meta-analyses classified calcium-supplemented women based on their risk status for preeclampsia, baseline calcium diet status, dosage, and the economic status of the countries in which the studies were conducted (Table 1).

**Table 1:** Characteristics of the included systematic reviews and meta-analyses, 2023.

Author	Year of publication	No primary article	Experimental	Control	Sample size	Status of women
Calcium supplementation despite the risk status of the pregnant women						
Imdad and Bhutta (40)	2012	15	8,231	8,259	16,490	Unknown status
Tang et al. (28)	2014	10	12,399	12,388	24,787	Unknown status
Li-bin An et al. (10)	2015	4	7,252	7,272	14,524	Unknown status
Win Khaing et al. (30)	2017	16	12,876	13,060	25,936	Unknown status
G. J. Hofmeyr et al. (5)	2018	13	7,851	7,879	15,730	Unknown status
Xiaotong Sun et al. (27)	2019	25	13,896	13,869	27,765	Unknown status
Christina Oh et al. (21)	2020	3	660	664	1,324	Unknown status
Mai-Lei Woo et al. (29)	2022	31	NR	NR	17,915	Unknown status
Risk status for preeclampsia						
Tito Silvio P. et al. (26)	2012	3	162	184	346	High risk
Tang et al. (28)	2014	4	4,320	4,345	8,665	High risk
G. J. Hofmeyr et al. (5)	2018	5	281	306	587	High risk
Win Khaing et al. (30)	2017	8	1,140	1,160	2,300	High risk
Xiaotong Sun et al. (27)	2019	12	797	820	1,617	High risk
Tippawan L. et al. (31)	2022	13	NR	NR	26,021	High risk
Dexin Chen et al. (32)	2022	3	99	100	199	High risk
Mai-Lei Woo et al. (29)	2022	17	1,540	2,121	3,661	High risk
José Villar et al. (9)	2000	6	3,146	3,161	6,307	Low risk
Tito Silvio P. et al. (26)	2012	7	5,535	5,524	11,059	Low risk
Tang et al. (28)	2014	6	8,049	8,043	16,092	Low risk
Win Khaing et al. (30)	2017	8	12,876	13,060	25,936	Low risk
G. J. Hofmeyr et al. (5)	2018	8	7,570	7,573	15,143	Low risk
Xiaotong Sun et al. (27)	2019	13	13,119	1,305	14,424	Low risk
Mai-Lei Woo et al. (29)	2022	13	8,423	8,361	16,784	Low risk
Baseline calcium dietary level status						
José Villar et al. (9)	2000	6	907	935	1842	Low baseline
Tito Silvio P. et al. (26)	2012	6	5,958	5,096	11,054	Low baseline
Imdad and Bhutta (40)	2012	10	5,711	5,727	11,438	Low baseline
Tang et al. (28)	2014	6	5,272	5,262	10,534	Low baseline
G. J. Hofmeyr et al. (5)	2018	8	5,331	5,347	10,678	Low baseline
Mai-Lei Woo et al. (29)	2022	24	7,266	7,784	15,050	Low baseline
Tito Silvio P. et al. (26)	2012	6	4,815	4,826	9,641	Adequate baseline
G. J. Hofmeyr et al. (5)	2018	4	2,505	2,517	5,022	Adequate baseline
Mai-Lei Woo et al. (29)	2022	6	2,697	2,698	5,395	Adequate baseline
Tang et al. (28)	2014	2	4,605	4,602	9,207	Unknown
G. J. Hofmeyr et al. (5)	2018	1	15	15	30	Unknown
Level of calcium supplementation dose						
Xiaotong Sun et al. (27)	2019	16	404	445	849	High
Mai-Lei Woo et al. (29)	2022	19	8,249	8,447	16,696	High
Dexin Chen et al. (31)	2022	13	12,942	12,839	25,781	High
Xiaotong Sun et al. (27)	2019	5	137	137	274	Moderate
Dexin Chen et al. (31)	2022	3	678	695	1,373	Moderate
Xiaotong Sun et al. (27)	2019	4	236	235	471	Low
Mai-Lei Woo et al. (29)	2022	12	2035	1714	3,749	Low
Dexin Chen et al. (31)	2022	13	1,498	1,498	1,498	Low
Economic status of the countries						
Win Khaing et al. (30)	2017	12	10,253	10,415	20,668	Developed
Xiaotong Sun et al. (27)	2019	4	4,508	4,526	10,034	Developed
Aamer Imdad et al. (33)	2011	10	5,697	5,708	11,405	Developing
Win Khaing et al. (30)	2017	4	2,641	2,645	5,286	Developing
Xiaotong Sun et al. (27)	2019	21	11,278	11,252	22,530	Developing

### Effects of calcium supplementation on preeclampsia

**Figure 2:**
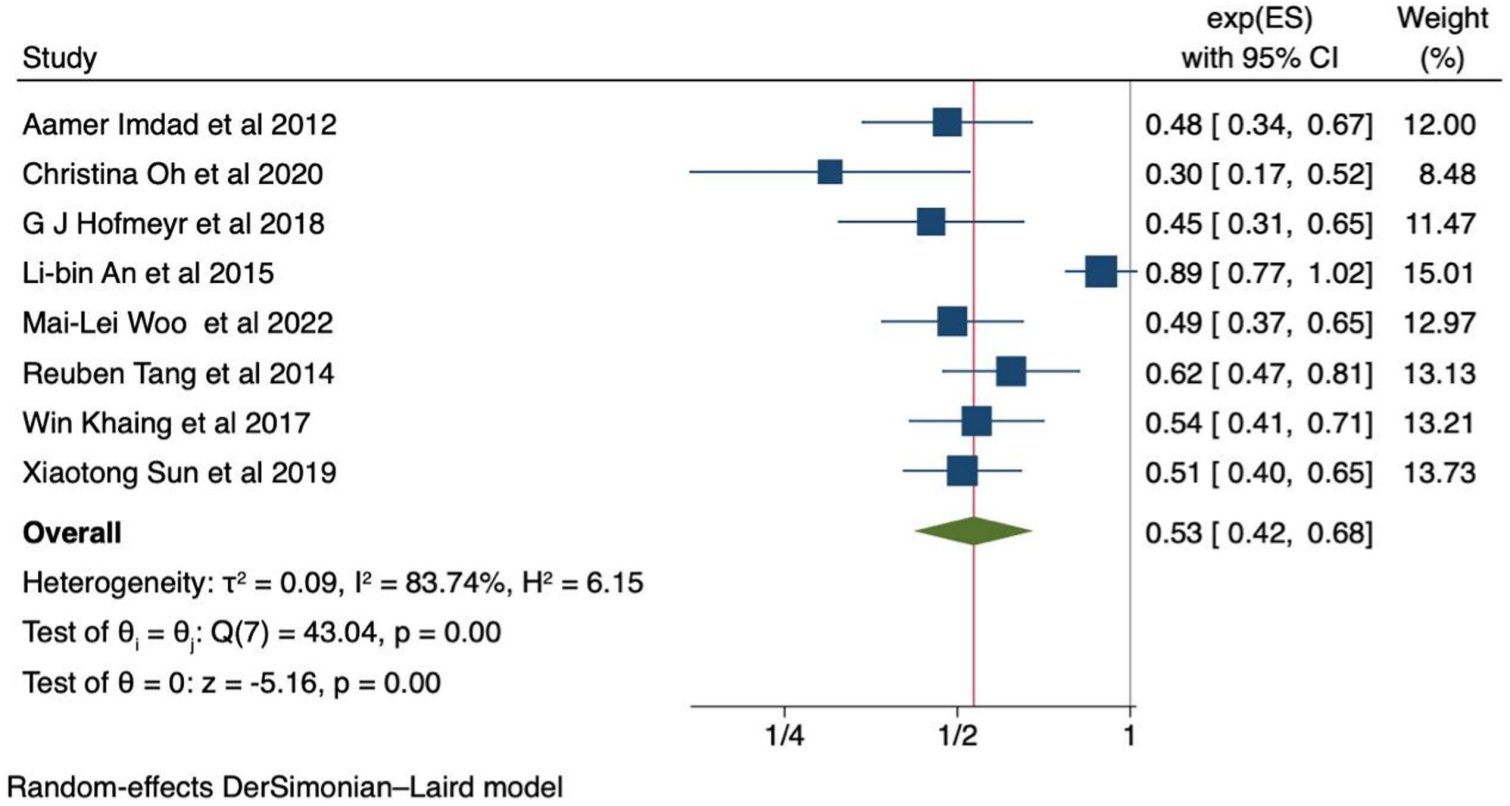
Forest plot of calcium supplementation and preeclampsia.

Using eight systematic review and meta-analysis studies, the effects of calcium supplementation on the reduction of preeclampsia incidence were reported, regardless of the risk for preeclampsia, baseline calcium diet status, and dose. The results revealed a significant reduction in the risk of preeclampsia in women receiving calcium supplementation [0.53 (95% CI: 0.42, 0.68)] (Figure 2). The level of heterogeneity was high, as evidenced by an I^2^ value of = 83.74%. Due to publication bias (*p* value less than 0.0001), a trim-and-fill analysis was conducted. However, differences in the results were not seen. The Galbraith and funnel plots are also available in Supplementary Figures S1, S2.

### Effects of calcium supplementation based on risk status for preeclampsia

**Figure 3:**
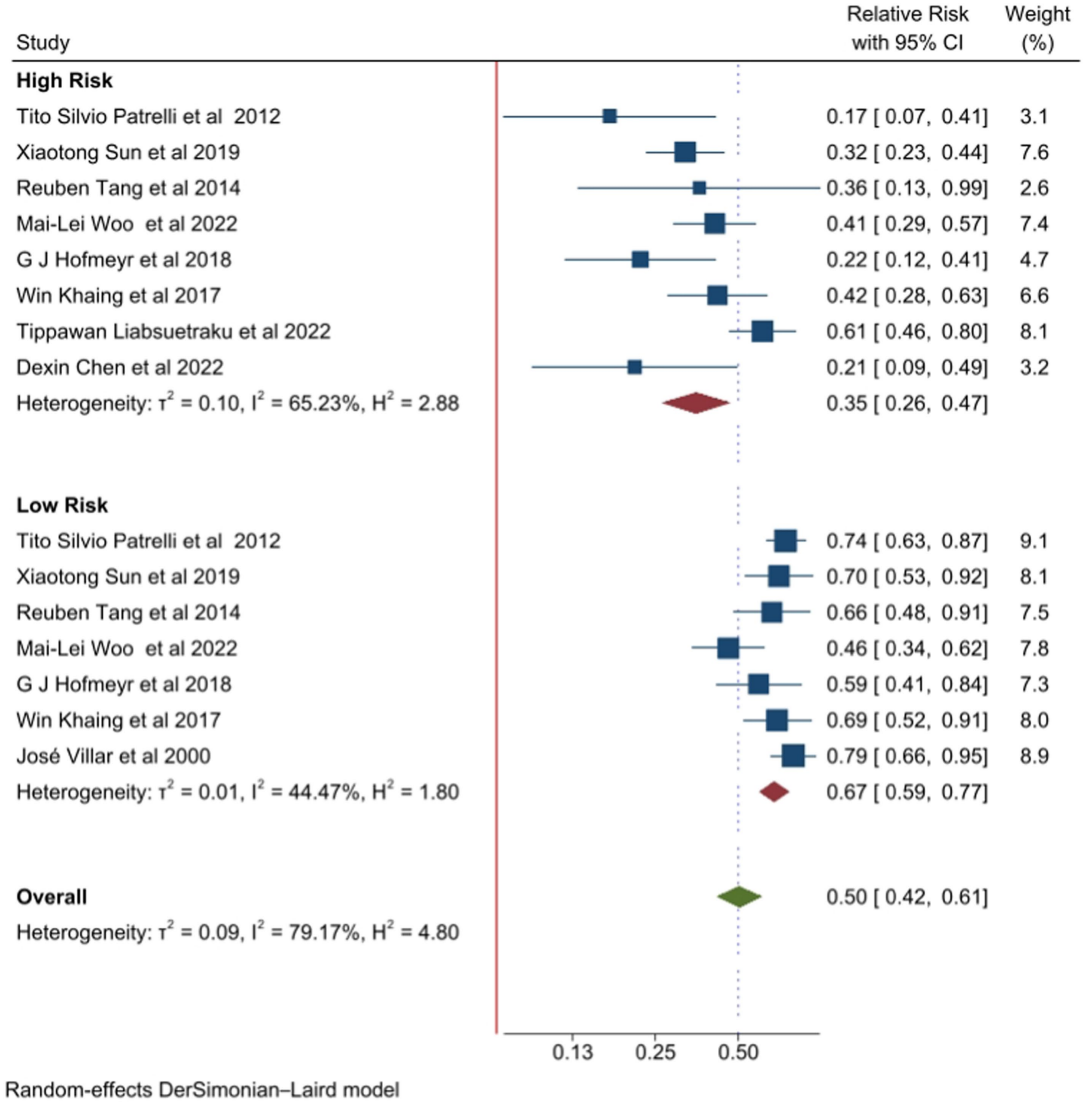
Forest plot of calcium supplementation and preeclampsia based on risk status.

Calcium supplementation in high-risk pregnancies was evaluated in eight studies (5, 26–32). The pooled relative risk showed a 65% reduction in the incidence of preeclampsia [RR 0.35 (95% CI: 0.26, 0.47)] (Figure 3). On the other hand, in seven studies, calcium supplementation in low-risk pregnancies showed 33% of reduction of preeclampsia [RR 0.67 (95% CI: 0.59, 0.77)] (Figure 3). We conducted an Egger’s test to evaluate the presence of publication bias, and the obtained *p*-value of 1 indicates that there is no evidence of publication bias. The Galbraith and funnel plots are available in Supplementary Figures S3, S4.

### Effects of calcium supplementation based on baseline dietary status

**Figure 4:**
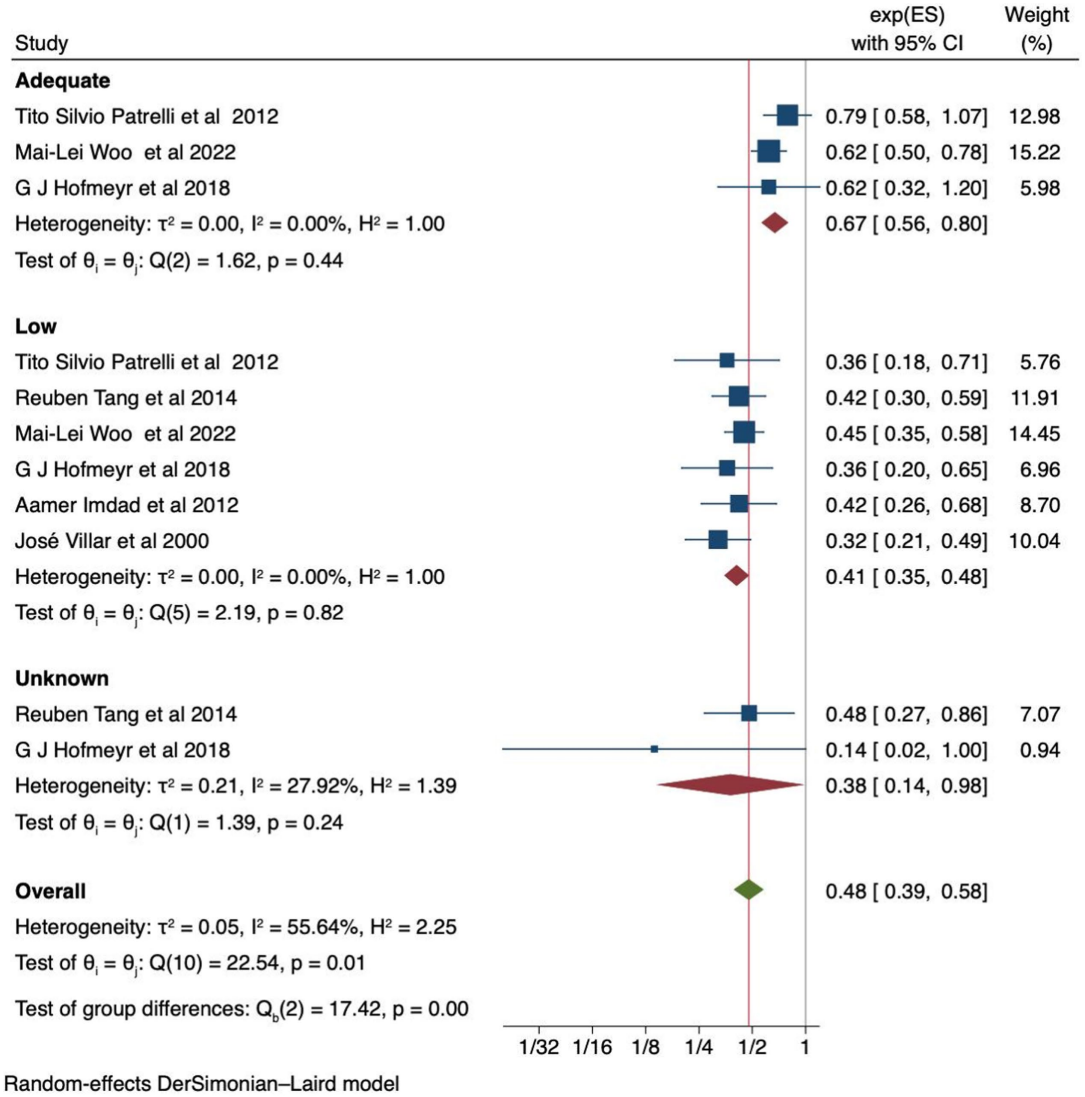
Forest plot of calcium supplementation and preeclampsia based on baseline calcium dietary status.

A pooled analysis of six studies found that women with a low baseline diet experienced a 59% reduction in the risk of developing preeclampsia with calcium supplementation [RR 0.41 (95% CI: 0.35–0.48)] compared to those who received a placebo, without heterogeneity between the studies (I^2^ = 0.0%). Similarly, calcium supplementation in women with an adequate baseline calcium level, as reported by three studies, was found to be associated with a 33% reduction in the risk of preeclampsia [RR 0.67 (95% CI: 0.56, 0.80)] without moderate heterogeneity (I^2^ = 0.0%), compared to placebo (Figure 4). Additionally, the Egger’s test yielded a p-value of 1, indicating no publication bias. The Galbraith and funnel plots are available in Supplementary Figures S5, S6, respectively.

### Effects of calcium supplementation based on dose

**Figure 5:**
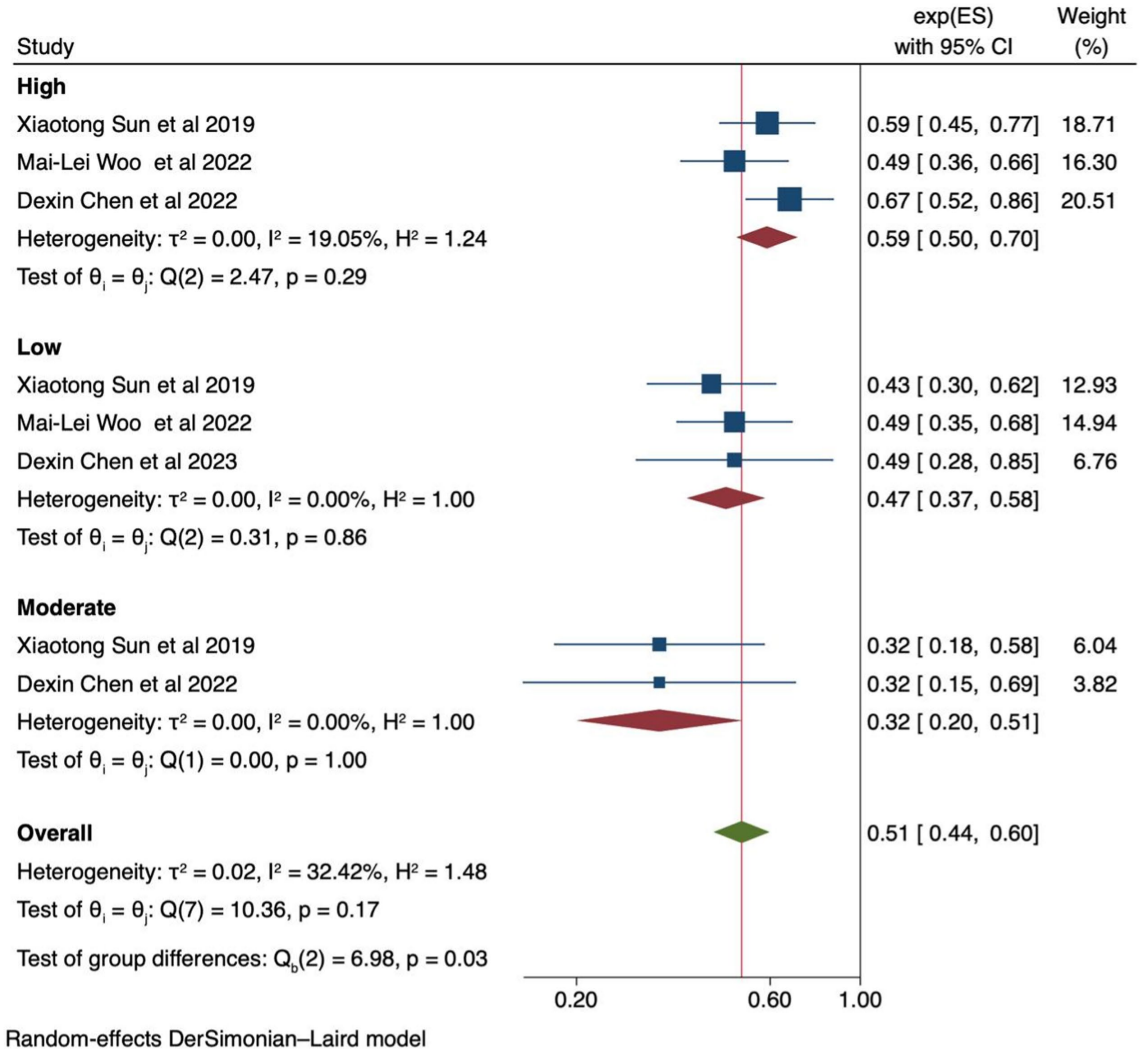
Forest plot of calcium supplementation and preeclampsia based on supplemented calcium dose level.

Sun et al. (27) and Chen et al. (32) classified calcium supplementation into three categories: high, moderate, and low doses, while Mai-Lei et al. (29) classified it into high and low. The pooled analysis of high-dose supplementation showed a steadily decreasing incidence of preeclampsia [RR 0.59 (95% CI: 0.51–0.69)]. The level of heterogeneity was low (I^2^ value of 19.05%). The forest plot is available in Figure 5. Furthermore, the Egger’s test yielded a *p*-value of 1. The Galbraith and funnel plots are available in Supplementary Figures S7, S8, respectively.

### Effects of calcium supplementation based on the economic status of the countries

**Figure 6:**
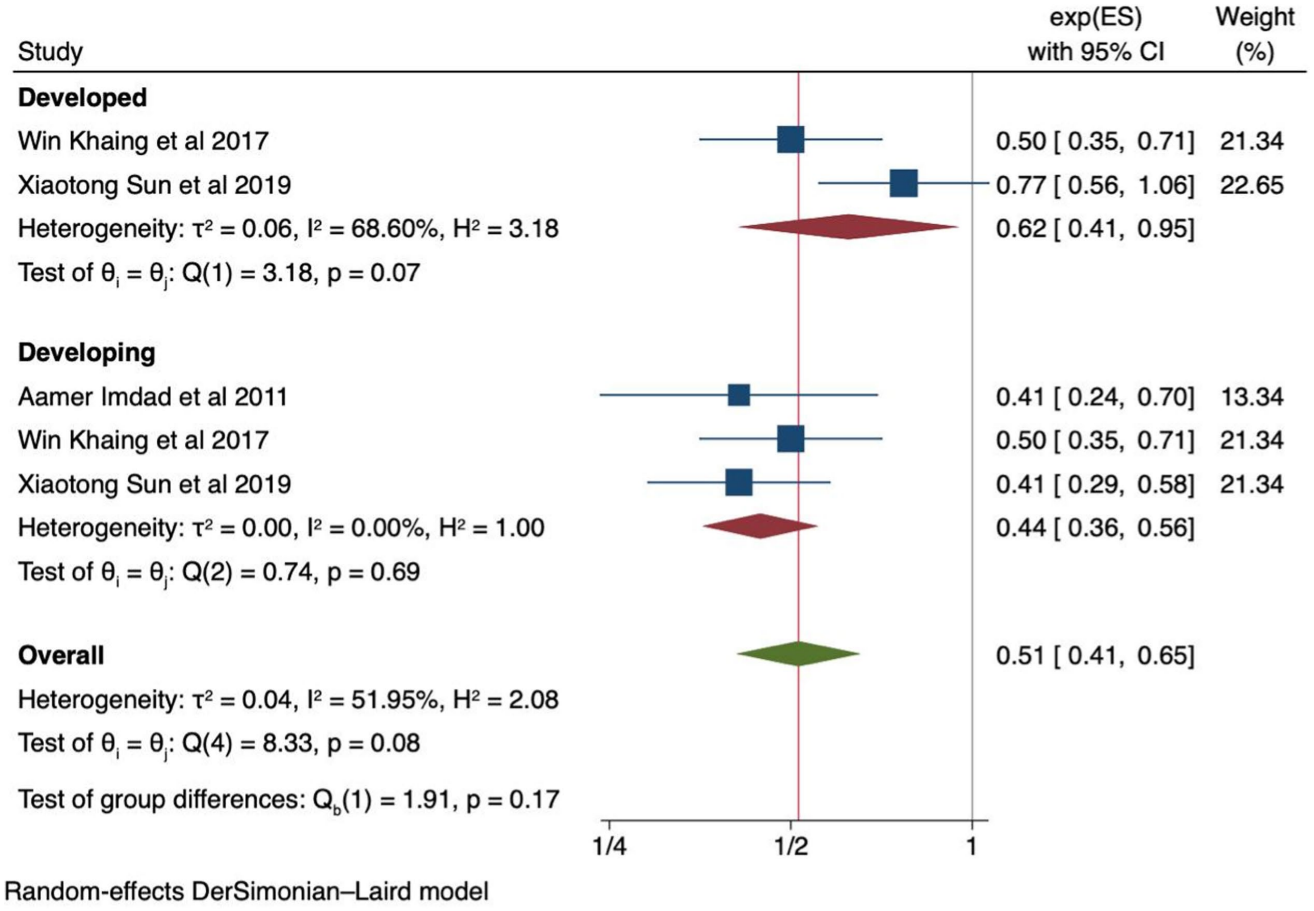
Forest plot of calcium supplementation and preeclampsia based on economic status economic status of the countries.

We conducted a subgroup analysis based on the economic status of the countries using five studies (27, 30, 33). In developed countries, calcium supplementation reduced the risk of preeclampsia by 38% [RR 0.62 (95% CI: 0.41, 0.95)]. In developing countries, a 56% reduction was noted [RR 0.44 (95% CI: 0.36, 0.56)] without heterogeneity (I^2^ = 00%) (Figure 6). Additionally, the Egger’s test yielded a *p*-value of 1, and funnel plots are also available in the Supplementary Figure S9.

### Sensitivity analysis

A leave-one-out sensitivity analysis was performed to identify the impact of each individual study on the overall pooled effect. The results of this sensitivity analysis showed that the pooled finding was not dependent on a single study (Supplementary Tables S4–S6).

## Discussion

Calcium supplementation to prevent preeclampsia is recommended by different guidelines (16, 34). However, there has been no prior umbrella review on using calcium supplementation to prevent preeclampsia. An umbrella review of eight systematic reviews and meta-analyses showed a significant reduction (47%) in the incidence of preeclampsia with the use of calcium supplements compared to control groups. Furthermore, it was observed that calcium supplementation significantly reduced the risk of preeclampsia in both high-risk women (65%) and low-risk women (33%) compared to placebo group.

In this umbrella review, calcium supplementation was associated with a 47% reduction in the incidence of preeclampsia among pregnant women. With the exception of one meta-analysis of multicenter randomized controlled trials, which reported an 11% reduction (10), the remaining seven meta-analyses included in this review consistently demonstrated a significant reduction in preeclampsia incidence in the calcium supplementation group compared to the control or placebo groups (5, 35). The discrepancy in findings may be due to the majority of the included primary studies being from developed or middle-upper income countries (10). Additionally, out of the four studies used for the meta-analysis, only one indicated that pregnant women had a low-calcium diet (10). However, the baseline calcium level is a crucial factor in reducing the incidence of preeclampsia with calcium supplementation.

In the current review, the pooled relative risk of preeclampsia in high-risk women with calcium supplementation compared to placebo reduced the risk of preeclampsia by 65% [0.35 (95% CI: 0.26, 0.47)] and I^2^ = 65.23. Although the effect was less pronounced in low-risk women, calcium supplementation still demonstrated a significant reduction in the incidence of preeclampsia (RR 0.67 95% CI: 0.59, 0.77). All the meta-analyses used in this umbrella review, both for high- and low-risk subgroup analyses, agreed on the reduction of preeclampsia incidence with calcium supplementation compared to placebo. However, the high level of heterogeneity observed among high-risk women for preeclampsia might be due to differences in risk definitions, variations in calcium dosage, and discrepancies in the timing of supplementation initiation among the primary studies included in the meta-analysis. Incorporating calcium supplementation into the guidelines for antenatal care, with special attention to high-risk pregnancies, may help to reduce maternal morbidity and mortality.

Recommended calcium intake for pregnant women varies between 900 and 1,200 mg/day, depending on the country (36, 37). However, actual intake among pregnant women often falls short of these recommendations, particularly in developing countries and even in developed countries where calcium-rich foods are readily available and affordable (19, 38). Furthermore, inconsistent results have been observed in systematic reviews and meta-analyses, with no effects in the prevention of preeclampsia on women with adequate calcium serum levels. However, a study conducted by Mai-Lei et al. showed a reduced incidence of preeclampsia. This umbrella review supports their finding that calcium supplementation in women with adequate baseline calcium levels has an effect on the prevention of preeclampsia. This finding suggests a potential need for increased calcium intake during pregnancy or that preeclampsia may involve multiple systems despite normal serum calcium levels. This can be explained by the significant physiological, metabolic, and hormonal changes that occur during pregnancy, which increase calcium demand (10, 11). One key factor is the heightened calcium demand of the developing fetus and growing placenta. This leads to increased calcium transfer from the mother to the fetus, depleting maternal calcium levels and heightening the risk of preeclampsia (11).

We found that calcium supplementation in women with low baseline calcium levels significantly reduced the risk of preeclampsia compared to placebo. Inadequate calcium intake, defined as consuming less than 600 mg daily, is associated with an increased incidence of hypertensive disorders during pregnancy (39). Moreover, the incidence of preeclampsia decreased significantly in the supplemented group in developing countries (56%) compared to developed countries (38%). This may explain the association between low calcium levels and hypertension (7, 8). Therefore, in regions where the diet is traditionally low in calcium and in developing countries, the recommendation of calcium supplementation during pregnancy is a safe approach. The authors also support the WHO recommendation of calcium supplementation as part of antenatal care in populations with low calcium intake (15).

We also found that high-dose and low-dose calcium supplementation have different effects on decreasing the incidence of preeclampsia. However, no strong signals indicated which group of women was more likely to benefit or be harmed, or which type of administration maximized the effect. Our findings do not suggest any superiority of high-dose over low-dose calcium supplementation. It should be noted that the study conducted by Chen et al. (22) used high-dose calcium supplementation only in high-risk pregnant women and low-dose in low-risk women, which may have influenced the results. High-dose calcium poses challenges in terms of cost, transportation, and storage. Low-dose calcium is equally effective as high-dose supplementation when initiated before 20 weeks of gestation (21). Lower dosages are recommended by the WHO (1.5–2.0 g/day) (16), and the fortification of staple foods with calcium could be considered for populations with low-calcium diets (21).

We categorized the outcomes based on various factors to provide a comprehensive perspective on calcium supplementation. This thorough inclusion of studies enhances the breadth of our review and offers a more detailed understanding of the efficacy of calcium supplementation in preventing preeclampsia.

Our findings should be considered in light of the limitations present in the included studies. The optimal timing of calcium supplementation for the prevention of preeclampsia may not be fully elucidated in the included studies. Variations in the timing and dosage of calcium supplementation across studies could introduce additional heterogeneity and affect the effect estimates. Furthermore, variations in the definition of preeclampsia across studies can lead to inconsistencies in pooled analyses, especially when combining data from trials with different control groups, such as placebo, no treatment controls, and alternative agents used. Additionally, the definition of risk varied between the individual studies and the pooled analysis, leading to potential inconsistencies in the findings. A few primary studies in the meta-analysis included co-supplementation of Vitamin D, which could be a potential source of heterogeneity.

## Conclusion

In conclusion, our findings indicate that daily calcium supplementation during pregnancy may be an effective strategy to prevent preeclampsia. The beneficial effect is more pronounced in women with low baseline calcium intake, those at high risk for preeclampsia, and those in developing nations. Considering the guidelines provided by the WHO, calcium supplementation can help reduce the incidence of preeclampsia and its associated complications. Furthermore, fortifying staple foods with calcium will be vital for populations with low-calcium diets.

## Data Availability

The original contributions presented in the study are included in the article/Supplementary material, further inquiries can be directed to the corresponding author.
