# Supplementary material for: Effects of calcium supplementation on the prevention of preeclampsia: an umbrella review of systematic reviews and meta-analyses
Source: Front Med (Lausanne). 2025 Mar 5;12:1434416. doi: 10.3389/fmed.2025.1434416 (PMC11922079; doi:10.3389/fmed.2025.1434416)
Supplement: Supplementary file 1 [file Supplementary_file_1.docx]

**Table** S**1:** Search strategy

| **N** |  | **Search terms** |
| --- | --- | --- |
| 1 |  | Pre-Eclampsia[Mesh] |
| 2 |  | preeclampsia |
| 3 |  | preeclamp* |
| 4 |  | pre-eclamp* |
| 5 |  | Pregnancy-induced hypertension |
| 6 |  | Hypertensive disorder during preganncy |
| 7 |  | 1 OR 2 OR 3 OR 4 OR 5 OR 6 |
| 8 |  | Calcium |
| 9 |  | Ca++ |
| 10 |  | Calcium[Mesh] |
| 11 |  | 8 OR 9 OR 10 |
| 12 |  | Systematic review and Meta anlsysis |
| 13 |  | 7 AND 11 AND12 |

Table S2: Quality assessment of the studies included in the umbrella review

| No | Author | Publication Year | Quality of review |
| --- | --- | --- | --- |
|  | Jose Villar et al | 2000 | Moderate quality review |
|  | Aamer Imdad et al | 2012 | Moderate quality review |
|  | Tito Silvo et al. | 2012 | Low quality review |
|  | Reuben Tang et al | 2014 | Low quality review |
|  | Li-bin An et al | 2015 | High quality review |
|  | Win Khaing et al | 2017 | High quality review |
|  | G J Hofmeyr et al | 2018 | High quality review |
|  | Xiaotong Sun et al | 2019 | Moderate quality review |
|  | Christina Oh et al | 2020 | Moderate quality review |
|  | Mai-Lei Woo et al | 2022 | Moderate quality review |
|  | Tippawan L. et al | 2022 | Moderate quality review |
|  | Dexin Chen et al | 2023 | Moderate quality review |

High (no or non-critical weakness), moderate (more than one critical weakness but no critical flaws), low (one critical flow with or without non-critical weaknesses), and critically low (more than one critical flaw with or without non-critical weaknesses)

| Study | Meta-analysis studies | | | | | | | | | | |
| --- | --- | --- | --- | --- | --- | --- | --- | --- | --- | --- | --- |
|  | Aamer  Imdad et al 2012 | Reuben Tang et al 2014 | | | Li-bin An et al. 2015 | Win Khaing et al 2017 | G J Hofmeyr et al 2018 | Mai-Lei Woo et al  2022 | Christina Oh et al 2020 | Fanny K. F.1 et al 2020 | Xiaotong Sun et al  2019 |
| Crowther 1999 | * | | * | * | | * | * | * |  |  | * |
| Levine 1997 | * | | * | * | | * |  | * |  |  | * |
| CPEP 1997 |  | |  |  | |  | * |  |  |  |  |
| Purwar 1996 | * | | * |  | | * | * | * |  |  | * |
| L-Jaramillo 1989 | * | |  |  | |  | * | * |  |  |  |
| L-Jaramillo 1997 | * | | * |  | | * | * | * | * |  | * |
| L-Jaramillo 1990 | * | |  |  | | * | * | * |  |  | * |
| S-Ramos 1994 | * | | * |  | | * | * | * |  |  | * |
| Wanchu 2001 | * | |  |  | |  |  |  |  |  |  |
| Taherian 2002 | * | |  |  | |  |  | * |  | * | * |
| Kumar 2009 | * | | * |  | |  | * | * | * | * | * |
| Belizan 1991 | * | | * | * | | * | * | * | * |  | * |
| Villar 2006 | * | | * | * | | * |  | * |  |  | * |
| Villar 1990 | * | |  |  | | * | * | * |  |  | * |
| Villar 1987 | * | |  |  | |  | * |  |  |  |  |
| Niromanesh 2001 | * | | * |  | | * | * | * |  | * | * |
| Nenad 2011 |  | | * |  | | * |  |  |  |  | * |
| Almanrte 1998 |  | |  |  | | * |  |  |  |  |  |
| Bassaw 1998 |  | |  |  | | * |  | * |  |  |  |
| Wanchu 2001 |  | |  |  | | * |  | * |  |  | * |
| Aghamohammadi 2015 |  | |  |  | | * |  |  |  | * | * |
| Almirante 1998 |  | |  |  | | * |  | * |  |  | * |
| WHO 2006 |  | |  |  | |  | * |  |  |  |  |
| Dizavandil 1998 |  | |  |  | |  |  | * |  |  |  |
| Souza 2014 |  | |  |  | |  |  | * |  |  |  |
| Karamali 2015 |  | |  |  | |  |  | * |  |  |  |
| Samimi 2016 |  | |  |  | |  |  | * |  | * | * |
| Marya 1987 |  | |  |  | |  |  | * |  |  | * |
| Ito 1994 |  | |  |  | |  |  | * |  |  |  |
| Cong 1995 |  | |  |  | |  |  | * |  |  |  |
| Herrara 1998 |  | |  |  | |  |  | * |  |  | * |
| Rogers 1999 |  | |  |  | |  |  | * |  |  |  |
| Rumiries 2006 |  | |  |  | |  |  | * |  |  |  |
| Herrera 2006 |  | |  |  | |  |  | * |  |  | * |
| Azami 2017 |  | |  |  | |  |  | * |  |  |  |
| Hofmeyer 2019 |  | |  |  | |  |  | * |  | * |  |
| Carole 2019 |  | |  |  | |  |  |  |  | * |  |
| Khan 2013 |  | |  |  | |  |  |  |  | * | * |
| Asemi 2012 |  | |  |  | |  |  |  |  |  | * |
| Cong 1993(3 studies) |  | |  |  | |  |  |  |  |  | * |

Table S3: Lists of primary studies included in meta-analysis of included studies

Figure S1: Galbraith plot of heterogeneity test among the studies conducted calcium supplementation and preeclampsia

Figure S2: Funnel plot of publication bias test among the studies conducted on calcium supplementation and preeclampsia

Figure S3: Galbraith plot of heterogeneity test among the studies conducted calcium supplementation on based on the risk status for preeclampsia

Figure S4: Funnel plot of publication bias test the studies conducted calcium supplementation based on risk status for preeclampsia

Figure S5: Galbraith plot of heterogeneity test among the studies conducted calcium supplementation on based on the baseline dietary status of calcium

Figure S6: Funnel plot of publication bias test the studies conducted women calcium supplementation based on the baseline dietary status of calcium

Figure S7: Galbraith plot of heterogeneity test among the studies conducted calcium supplementation on based on the supplemented calcium dose level

Figure S8: Funnel plot of publication bias test the studies conducted women calcium supplementation pregnant women based on supplemented dose level

Figure S9: Funnel plot of publication bias test the studies based on economic status of countries

**Sensitivity analysis results**

Table S4: Sensitivity analysis result of studies used to estimate the effect of calcium supplementation in reducing the risk of developing preeclampsia with unknown status

| Study omitted | Publication year | e coef. | 95% CI |
| --- | --- | --- | --- |
| Aamer Imdad et al | 2012 | 0.52 | 0.41 - 0.67 |
| Christina Oh et al | 2020 | 0.54 | 0.43 - 0.68 |
| G J Hofmeyr et al | 2020 | 0.53 | 0.41 - 0.67 |
| Li-bin An et al | 2015 | 0.49 | 0.44 - 0.55 |
| Mai-Lei Woo et al | 2022 | 0.52 | 0.41 - 0.67 |
| Reuben Tang et al | 2014 | 0.50 | 0.39 - 0.66 |
| Win Khaing et al | 2017 | 0.51 | 0.40 - 0.66 |
| Xiaotong Sun et al | 2019 | 0.51 | 0.40 - 0.67 |
| Combined | | 0.52 | 0.41 - 0.65 |

Table S5: Sensitivity analysis result of studies used to estimate the effect of calcium supplementation in reducing the risk of developing preeclampsia among high risk pregnant women

| Study omitted | Publication year | e coef. | 95% CI |
| --- | --- | --- | --- |
| Tito Silvio P. et al | 2012 | 0.37 | 0.41 - 0.50 |
| Xiaotong Sun et al | 2019 | 0.35 | 0.25 - 0.49 |
| Reuben Tang et al | 2014 | 0.35 | 0.25 - 0.47 |
| Mai-Lei Woo et al | 2022 | 0.33 | 0.23 - 0.47 |
| G J Hofmeyr et al | 2018 | 0.37 | 0.28 - 0.50 |
| Win Khaing et al | 2017 | 0.33 | 0.23 - 0.47 |
| Tippawan L. et al | 2022 | 0.32 | 0.26 - 0.41 |
| Dexin Chen et al | 2022 | 0.37 | 0.27 - 0.49 |
| Combined | | 0.35 | 0.26 - 0.47 |

Table S6: Sensitivity analysis result of studies used to estimate the effect of calcium supplementation in reducing the risk of developing preeclampsia among low risk pregnant women

| Study omitted | Publication year | e coef. | 95% CI |
| --- | --- | --- | --- |
| Tito Silvio P. et al | 2012 | 0.65 | 0.56 - 0.77 |
| Xiaotong Sun et al | 2019 | 0.67 | 0.58 - 0.78 |
| Reuben Tang et al | 2014 | 0.67 | 0.58 - 0.77 |
| Mai-Lei Woo et al | 2022 | 0.72 | 0.66 - 0.79 |
| G J Hofmeyr et al | 2018 | 0.68 | 0.59 - 0.78 |
| Win Khaing et al | 2017 | 0.67 | 0.57 - 0.77 |
| Jose Villar et al | 2000 | 0.65 | 0.56 - 0.74 |
| Combined | | 0.67 | 0.59 - 0.77 |
